# Understanding Air Quality Changes after Implementation of Mitigation Measures during a Pandemic: A Scoping Review of Literature in the United States

**DOI:** 10.4209/aaqr.220047

**Published:** 2022-11

**Authors:** Sara McElroy, Ambarish Vaidyanathan

**Affiliations:** 1 Oak Ridge Institute for Science and Education, Oak Ridge, TN USA and Climate and Health Program, National Center for Environmental Health, Centers for Disease Control and Prevention, Atlanta, GA 30341, USA; 2 Climate and Health Program, DEHSP, NCEH, CDC, National Center for Environmental Health, Centers for Disease Control and Prevention, Atlanta, GA 30341, USA

**Keywords:** COVID-19, Air pollution, Mitigation measures, Mobility, Public health interventions

## Abstract

Traffic-related emissions continue to be a significant source of air pollution in the United States (US) and around the globe. Evidence has shown that previous policies implemented to restrict-traffic flows have affected air pollution levels. Thus, mitigation strategies associated with the COVID-19 pandemic that modified population-level mobility patterns provide a unique opportunity to study air pollution change across the US. For instance, to slow the spread of the pandemic, state and local governments started implementing various mitigation actions, including stay-at-home directives, social distancing measures, school closures, and travel restrictions. This scoping review aimed to summarize the existing evidence about how air quality changed through mitigation practices throughout the pandemic in the US. We found 66 articles that fit our inclusion criteria. Generally, the consolidated results revealed that nitrogen dioxide (NO_2_) and carbon monoxide (CO) decreased across the country. Studies observed mixed directions and magnitudes of change for fine and coarse particulate matter (PM_2.5_, PM_10_), ozone (O_3_), and sulfur dioxide (SO_2_). Few articles tried to explain this notable heterogeneity in air quality changes by associating contextual factors, such as mobility, traffic flow, and demographic factors. However, all studies agreed that the change in air pollution was nonuniform across the US and even varied within a city.

## 1 INTRODUCTION

Traffic-related emissions are an important source of ambient air pollution to which the majority of the population is exposed to in the United States (US) and other parts of the world. On-road combustion sources are a major contributor of various air pollutants, including criteria pollutants such as nitrogen dioxide (NO_2_), carbon monoxide (CO), fine particulate matter (PM_2.5_), and ozone (O_3_) (Bradley *et al*., 1999, Russell *et al*., 2012). In the past, there has been evidence showing that strategies implemented to restrict traffic-related mobility have led to changes in air quality levels (Peel *et al*., 2010). For example, polices that were implemented to reduce traffic congestion during the Atlanta and Beijing Olympic Games, were found to be associated with reduced air pollutant concentrations (Friedman *et al*., 2001; Li *et al*., 2010). These strategies included 24-hour public transportation, alternative work hours and telecommuting for local businesses, banning of vehicles that failed emission standards, restricting private vehicles to drive every other day, and closures of areas to private vehicles (Balagas, 1996; Porter, 1997; The People’s Government of Beijing Municipality, 2009). The changes in population-level mobility during the COVID-19 pandemic has provided a similar opportunity to study how mitigation strategies have impacted air quality in the United States.

The World Health Organization (WHO) issued a public health emergency on January 30, 2020, when coronavirus, SARS-CoV-2, was starting to spread across the world (WHO, 2020; Bashir *et al*., 2020; Wu *et al*., 2020). The WHO declared the disease a global pandemic on March 11, 2020 (Cucinotta and Vanelli, 2020), soon followed by Centers for Disease Control and Prevention (CDC) emphasizing the evidence of community spread in the United States. Many major cities across the United States took actions to mitigate community spread and began implementing stay-at-home orders, lockdown measures, social distancing measures, travel restrictions, school closures, and promoting telecommuting and remote work options (Cohen and Kupferschmidt, 2020; Pepe *et al*., 2020; Schlosser *et al*., 2020).

These mitigation efforts and countermeasures for preventing the spread of the pandemic have brought about behavioral changes in our society. For instance, remote work and telecommuting options combined with stay-at-home directives have brought about shifts in mobility patterns. These personal behavioral modifications and community-level mitigation practices provide an opportunity to assess the relationship between interventions and their collective impact on air quality levels.

However, community-level mitigation policies vary significantly across states or even within counties in the US (Raifman *et al*., 2020). Researchers have assessed changes in air pollution, which reflect the impact of mitigation efforts on emission changes associated with various air pollution sources, and changes in standard daily activity patterns within cities by analyzing mobility, traffic, and economic information (Campbell *et al*., 2021; Heintzelman *et al*., 2021; Winchester *et al*., 2021). For instance, researchers have investigated the impact of stay-at-home orders on air quality levels under the assumption that changes in transportation patterns and mobility would lead to lower levels of traffic-related air pollutants. Of note, air pollutants such as NO_2_, O_3_, CO, coarse particulate matter (PM_10_) and PM_2.5_ are determinants of economic and human development (Venter *et al*., 2021). Primary sources of these air pollutants include transportation and other non-road combustion sources (Russell *et al*., 2012; Bradley *et al*., 1999; Manousakas *et al*., 2017).

We conducted this scoping review to summarize published scientific articles examining how air quality changed due to community-level pandemic mitigation measures within the US. This scoping review aims to summarize findings from published research exploring change in air pollution levels occurring in the period with COVID-19 mitigation practices (e.g., shelter-in-place directives). Specifically, we report on exposure data used, methodologies, the results of these assessments, contextual factors, mediators, and other studies outcomes. Our review summarizes the available evidence to provide an overview of the topic for researchers interested in linking measurable relationships between policies that reduce emissions and pollutant concentration.

Specifically, the review question is: how did ambient air pollution change from previous years to the period where COVID-19 restrictions were enacted in the US? Furthermore, how did this observed change in air pollution vary by geography and time? In addition, have studies explored contextual factors that can explain the relationship between air quality changes and COVID-19 mitigation measures?

## 2 METHODS

### 2.1 Study Design

We developed our scoping review using the methodology framework proposed by the Joanna Briggs Institute, built on the previous methodology proposed by Arksey and O’Malley (Arksey and O’Malley, 2005; Peters *et al*., 2015). This review was conducted utilizing the five steps recommended by Arksey and O’Malley (2005): 1) identifying a valid research question and defining search strategies, 2) identifying relevant studies, 3) study selection, 4) extracting and charting data, and 5) collating, summarizing, discussing, and reporting results.

### 2.2 Search Strategy

The leading search strategy aims to identify published scientific articles, or articles in preprint, in English. An initial limited search of MEDLINE (PubMed) and Google Scholar was executed to detect articles pertaining to this topic. These articles provided insights for text words that were included in titles, abstracts, and keywords. The information found from the preliminary search informed the development of a search strategy, including identified keywords and index terms that were tailored for each database. Since COVID-19 is a very recent development, we decided to constrict our scoping review to articles that have been published or are in preprint after the implementation of COVID-19 mitigation strategies (March 1, 2020, through October 1, 2021). The reference list of included studies was also assessed to find additional studies. The literature for this review was identified by searching the following online databases: PubMed, Web of Science, Embase, Global Health, Environmental Science Collection, Scopus, and Google Scholar. Our inclusion criteria included scientific articles published in English within the designated date range that discussed changes in air quality within the United States. We considered scientific commentaries, editorials, descriptive studies, comparison studies, exposure studies, and epidemiological study designs, including before and after studies, ecological studies, cross-sectional studies, cohort studies, case-control studies, spatiotemporal studies, and cluster analyses. Conference abstracts are not included in this study. The scope of this review does not include nonscientific publications, news articles, unpublished and gray literature, and systematic literature reviews. The complete list of search terms is located in Appendix A.

### 2.3 Identification and Selection of Relevant Studies

**Fig. 1.**
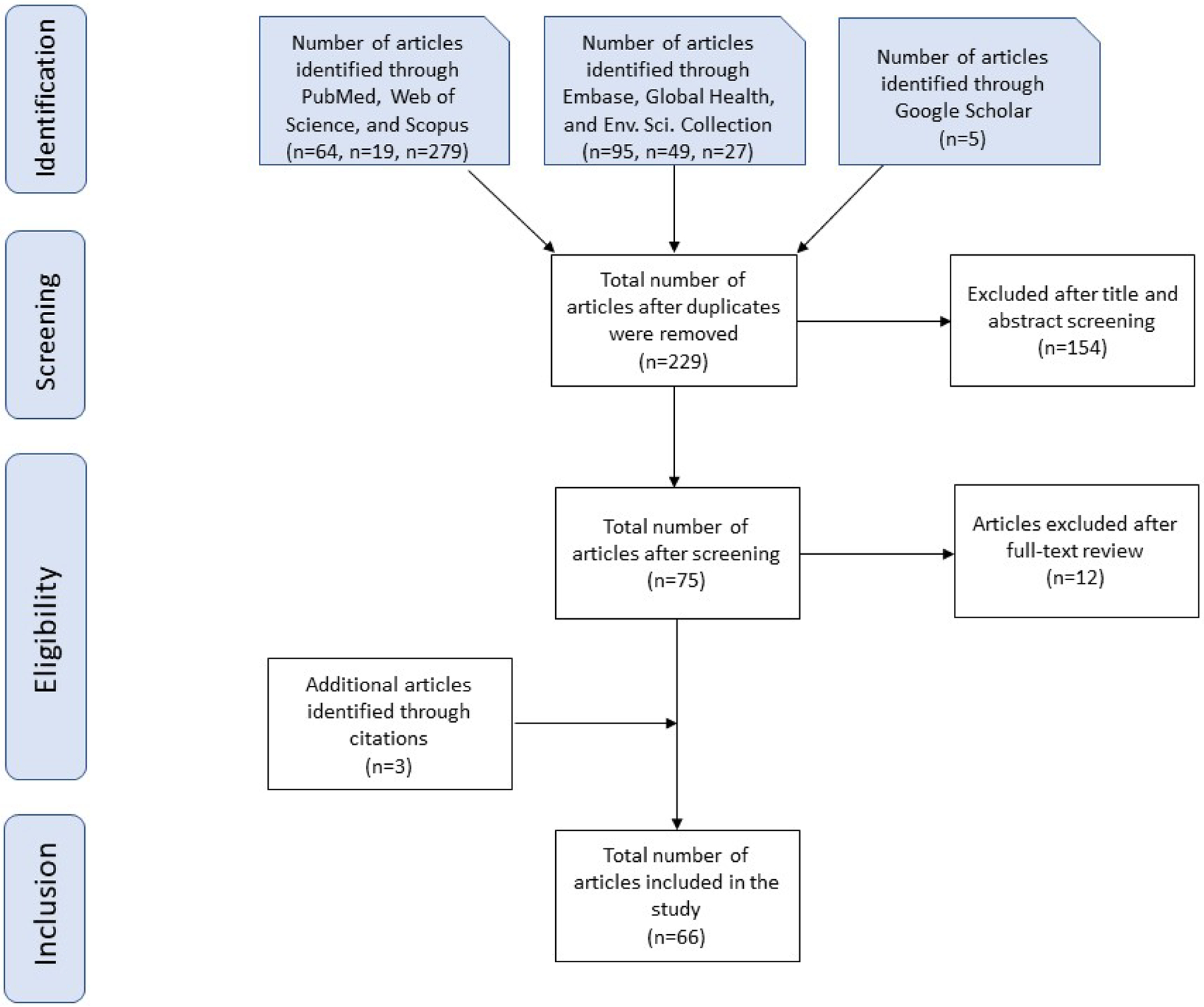
PRISMA Flow chart of scoping review inclusion and exclusion criteria.

The seven online databases were searched using the predetermined keywords. An example of these key words search is “air quality” OR “air pollution” OR “ambient exposure” AND “COVID-19*” OR “SARS Coronavirus 2 Infection” OR “SARS*” AND “US” OR “United States” If a question arose about the inclusion or exclusion of literature, an independent second researcher was brought in to make the final decision. Duplicate articles were eliminated. Fig. 1 depicts the process of our inclusion and exclusion of articles by a flow diagram per the Preferred Reporting Items for Systematic Reviews and Meta-Analyses (PRISMA) methodology (Moher *et al*., 2009).

### 2.4 Data Extraction

Data extracted from articles included in the scoping review were entered into an excel spreadsheet by a reviewer. Specific details about the populations, concept, context, and study methodologies of significance to the scoping review research question and aims were abstracted. More specifically, the author, journal’s name, study population, study location, study design, statistical analyses, source of exposure data, outcome, study period, covariates, main findings, and reviewer comments were included in the data abstracted.

### 2.5 Methodology Quality and Appraisal

Because our research aims were to summarize available evidence and identify gaps in the literature, we did not appraise the methodology quality or bias of the included articles, consistent with guidance on scoping review conduct (Peters *et al*., 2015).

### 2.6 Presentation of Results and Summarizing the Findings

Following our proposed research objectives, articles fell into three main categories. The first category entailed publications that only examined changes in air pollution between pre-and-during-COVID-19. The pre-COVID-19 period in the U.S. is defined as any time before March 1^st^, 2020, and the during-COVID-19 period is defined as any time after March 31^st^, 2020, to December 31^st^, 2021. The second group of articles examined changes across space and time by examining changes in air pollution across several geographic locations. The third group included contextual factors to explain the differences in air quality between pre-and current-COVID-19 periods. All results and statements regarding changes in air quality throughout the pandemic restrictions were abstracted from published information.

## 3 RESULTS

We identified 538 published articles; 309 articles were excluded as duplicates. After title and abstract screening were conducted for the remaining 229 articles, 154 were excluded because they did not fit our inclusion criteria. If the title indicated the study was performed outside the United States, involved examining COVID-19 mortality/case rates, studied indoor air pollution, or examined the biology of the virus, the article was excluded. A full-text review was implemented for the remaining 75 articles, 12 articles were excluded due to subject matter, or the article was a conference abstract. After examining citations from these articles, an additional three studies were found, resulting in a total of 66 scientific articles included in this scoping review (Fig. 1). The data abstraction table for the included articles in this scoping review can be found in Appendix B.

### 3.1 Literature Characteristics

Among the 66 included articles in this study, publication dates ranged from June 2020 to preprint articles that will be published in November of 2021. A little less than half the studies were published in 2020 (46%) compared to 2021 (54%). Included articles were conducted in a single city (n = 10, 15%), the continental US or multiple cities within the US (n = 14, 17%), one state in the US (n = 7, 9%), a US city and other international cities (n = 5, 8%), several countries including the US (n = 9, 15%) and globally (n = 21, 34%). The included articles were published in 29 different peer-review journals, with the most articles published in Science of the Total Environment (n = 12, 18%). Study periods ranged from 6 weeks to the entire year of 2020 (Table 1).

### 3.2 Air pollution Data

**Fig. 2.**
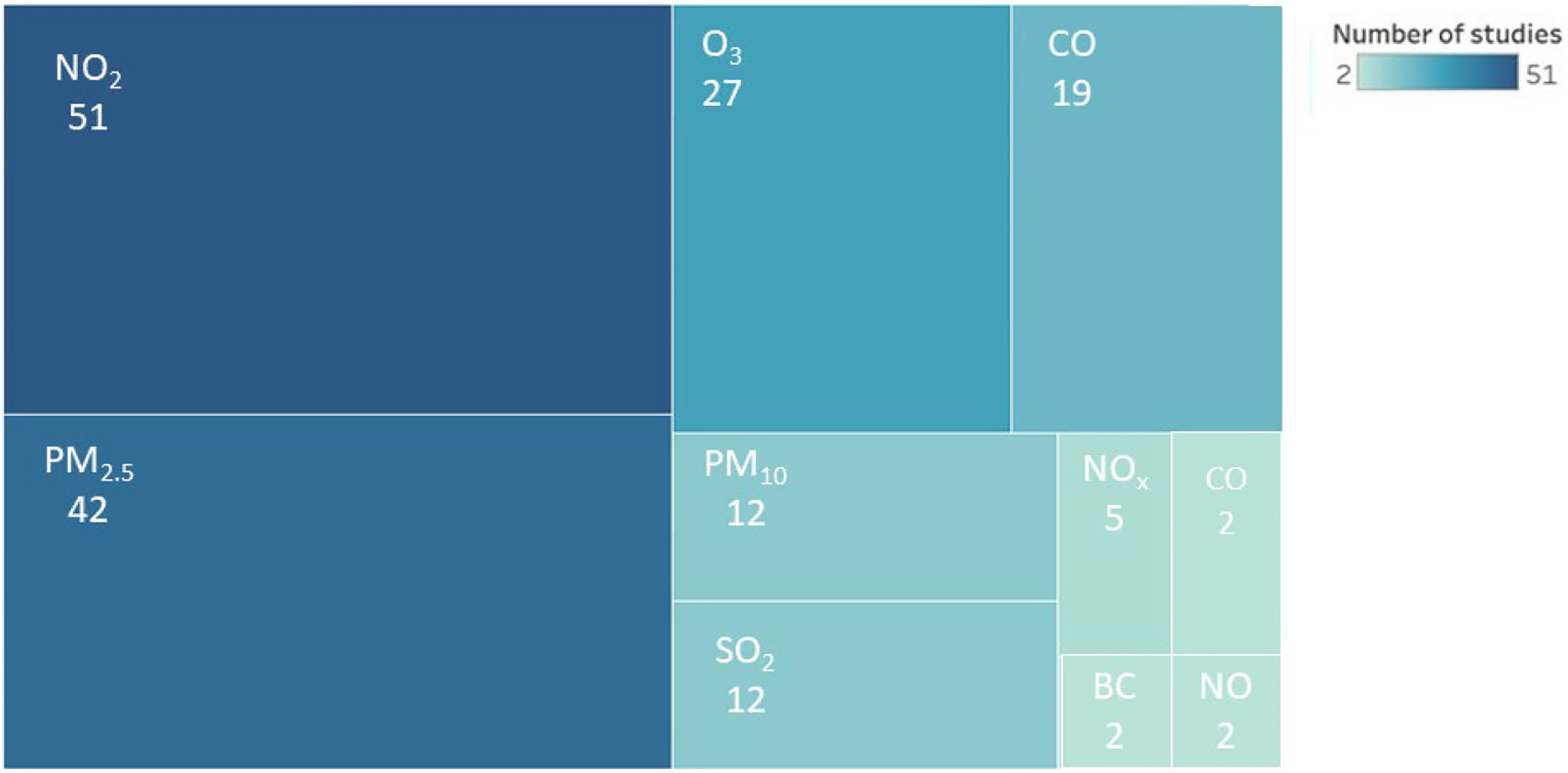
A thematic map of number of studies included in this scoping review, by air pollutants. Studies could examine more than one pollutant. The numbers reflect the total number of studies that examined a particular pollutant.

The included articles studied air pollutants or proxy measures of air quality including NO_2_ and/or other nitrogen oxides (NO_x_), CO, PM_2.5_, PM_10_, O_3_, aerosol optical depth (AOD, a proxy for particulates), black carbon (BC), and SO_2_. Forty-three studies included more than one air pollutants (65%). Twenty-three studies examined one air pollutant, which was either NO_2_, PM_2.5_, or CO_2_. The distribution of the number of studies that included each air quality measure can be seen in Fig. 2. Over half of the studies measured changes in air pollution concentrations, 19% measured changes in both concentrations and emissions, and 25% measured changes in vertical column density. There were 34 studies that retrieved air pollutant measurements from ground monitors (51%), 13 studies combined information from ground monitors and satellite data (20%), 14 studies relied on satellite data alone (21%), and five studies utilized mobile air pollution monitors (8%) to measure air pollutants. Articles which included ground monitor measurements retrieved those data from the US Environmental Protection Agency (EPA), AirNow (a partnership with the EPA), National Oceanic Atmospheric Administration (NOAA), National Park Service, NASA, CDC, and tribal, state, and local air quality agencies (https://www.airnow.gov/), Aerosol Robotic Network (AERONET), OpenAQ API, local county air quality measures, and the World Air Quality Index (WAQI) project. Remotely sensed data were obtained from multiple sources, which include the Tropospheric Monitoring Instrument (TROPOMI) onboard Sentinel-5 Precursor, the Ozone Monitoring Instrument (OMI) on Aura satellite, Moderate Resolution Imaging Spectroradiometer (MODIS) instrument on NASA’s Terra and Aqua satellites, the NOAA Suomi-NPP, and the Measure of Pollution in the Troposphere (MOPITT) on NASA’s Terra satellite (Fig. 2).

### 3.3 Study Design

Many of the included articles’ study design capitalized on the opportunity that arose from COVID-19 restrictions, which created a natural experiment, allowing for comparison of before and after periods (Bar *et al*., 2021; Campbell *et al*., 2021; Mendoza *et al*., 2021). Percent change methodology was utilized the most (n = 24) to quantify the change in air quality between a historical period and the COVID-19 period (See Fig. 4). Additional methodologies include unsupervised machine learning algorithms (Venter *et al*., 2020; Keller *et al*., 2021; Zhang *et al*., 2021), anomalies calculation (Zhang *et al*., 2020), time-lagged linear regression with ANCOVA and F-tests (Zangari *et al*., 2020), difference-in-difference quasi-experimental method (Brodeur *et al*., 2021), probability density functions (Mishra *et al*., 2021), two-sample t-tests (Berman and Ebisu, 2020; Jia *et al*., 2020; Chadwick *et al*., 2021; Elshorbany *et al*., 2021), non-parametric Wilcoxon rank-sum test (Hudda *et al*., 2020), regression discontinuity design (Hudda *et al*., 2020), robust differences with median and interquartile range (IQR) (Bekbulat *et al*., 2021) and general linear models (Connerton *et al*., 2020). Four articles were descriptive and did not perform any statistical analyses (Balasubramaniam *et al*., 2020; Hoang *et al*., 2021; Irfan *et al*., 2021; Mousazadeh *et al*., 2021). Ten studies were time-series analyses utilizing multivariate autoregressive models (Xiang *et al*., 2020), seasonal autoregressive integrated moving average with exogenous factors (SARIMAX) (Wong *et al*., 2021), Stochastic Time-Inverted Lagrangian Transport model (Turner *et al*., 2020), general additive model (GAM) (Liu *et al*., 2021a), moving averages (Liu *et al*., 2021b, 2021c), autoregressive moving averages models (ARIMA) (Winchester *et al*., 2021), principal component analysis (Savtchenko and Khayat, 2021), and mixed effect models (Ghosal and Saha, 2021). Lastly, one study performed a spatial cluster analysis using Getis-Ord (Gi*) spatial statistic (Straka *et al*., 2021).

### 3.4 Principal Findings

The change in ambient air quality from pre-COVID mitigation strategies was found to have increased, decreased, or stay the same depending on air pollutant and geographical location. There was a consensus that NO_2_ and CO decreased within the COVID-19 lockdown period, but the decreased level varied across geographical regions. Reductions in NO_2_ varied widely across studies from 18% to 63%. Also, many studies agreed that O_3_ increased in urban areas and decreased in rural areas throughout the policies implemented to stop the spread of COVID-19. Increases in O_3_ percent change ranged from 17% to 86%, and the decrease in O_3_ ranged from 1% to 10%. There were mixed results when examining PM_2.5_. Some studies found decreases in PM_2.5_, increases in PM_2.5_, and other studies found no variation from historical levels. Finally, two studies found no change in any air pollution criterion levels in 2020 than historical data. The effect sizes and results from each study can be seen in Appendix: Table S1.

**Fig. 3.**
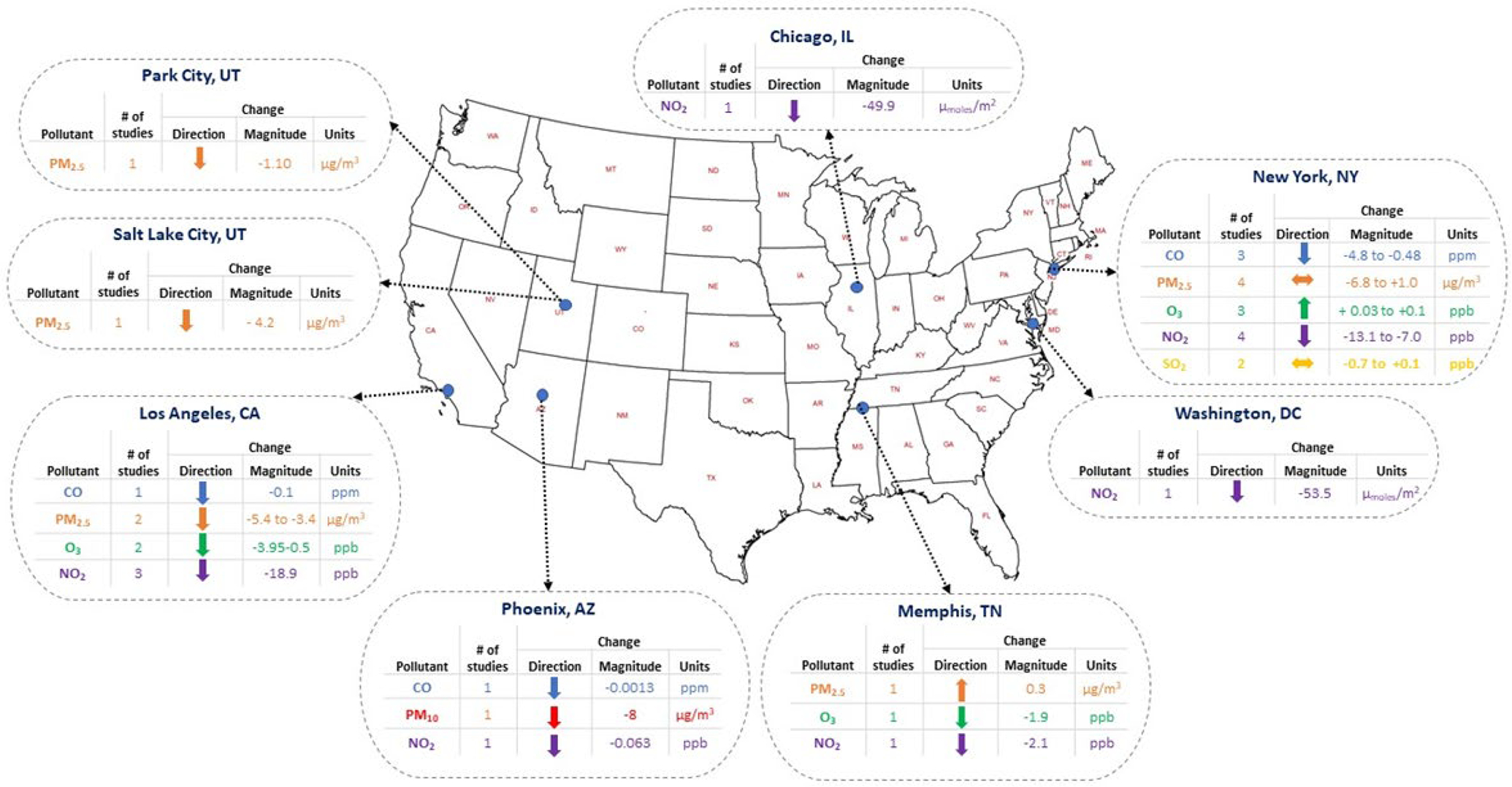
Location-specific summary of magnitude and direction of change in air pollutants.

**Fig. 4.**
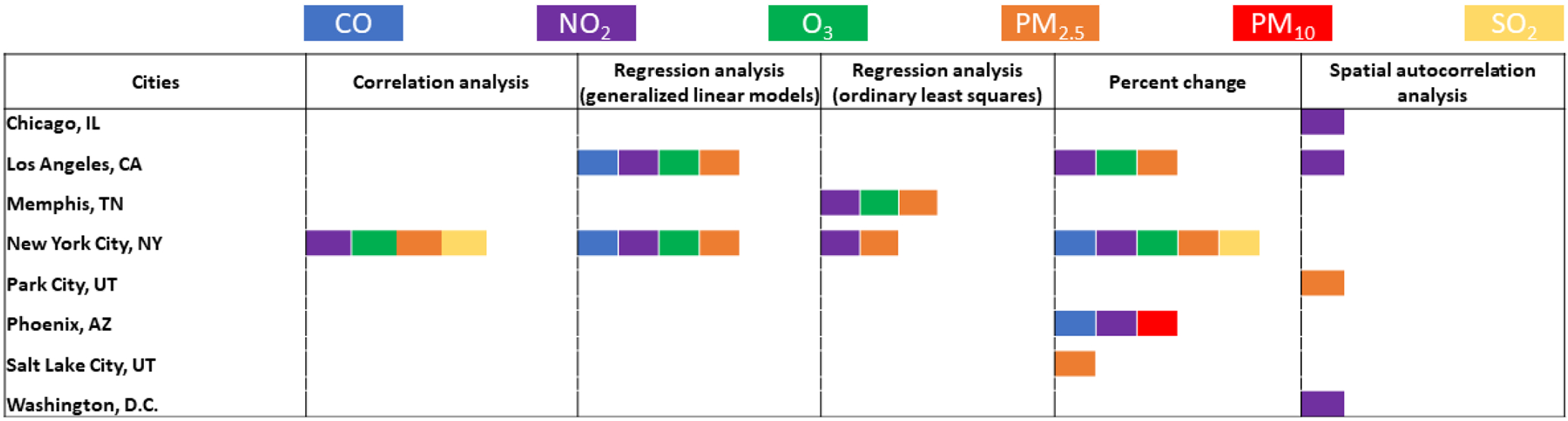
Methodologies utilized in this scoping review, by location and air pollutant.

City-specific results fit the general pattern observed from the overall findings. NO_2_ and CO level reductions were reported in all cities, with the most significant NO_2_ and CO emissions decrease in Los Angeles and New York City, respectively. PM_2.5_, O_3_, and SO_2_ levels varied across cities and within cities, with both observed increases and decreases of the air pollutant. The reduction of PM_2.5_ levels was the largest in Los Angeles, and the largest increase was recorded in New York City. O_3_ increased in New York City but decreased in Memphis and Los Angeles. Directions and magnitudes of air pollutant changes from city-specific studies can be seen in Fig. 3. Methodologies used in each of these studies can be seen in Fig. 4.

The sources of emission of NO_2_ consist of 90% from on-road and non-road combustion and biomass burning, and the remaining 10% primarily comes from chemical productions at power plants (Richter *et al*., 2004; Otmani *et al*., 2020). Studies that found a reduction in NO_2_ during the COVID-19 restrictions tied this reduction to both sources. The stay-at-home orders significantly reduced motor vehicle traffic, which resulted in a significant reduction of vehicle exhaust emissions (Archer *et al*., 2020; Chen *et al*., 2020; Connerton *et al*., 2020; Wang and Li, 2021). Furthermore, some factories were closed during the early stages of the pandemic, which curbed the production of NO_2_ (Wang and Li, 2021). CO reductions were consistent across studies, and these reductions were also linked to vehicle emissions, as vehicle traffic is the primary source of both NO_2_ and CO (Tanzer-Gruener *et al*., 2020). Moreover, NO_2_ and CO are primary pollutants; thus, changes in activity can be directly connected with emission levels.

Several hypotheses were proposed to explain why some studies did not observe a decrease in PM_2.5_ during the lockdown period. One such proposed reason was that the stay-at-home orders mainly affected individuals’ driving patterns. However, commercial vehicles (fueled primarily by diesel) and electricity demand (often met by burning of fossil fuels) remained unchanged. As a result, a reduction in PM_2.5_ might not be prominent (Archer *et al*., 2020). Secondly, PM_2.5_ is essentially a pollutant created from complex and nonlinear atmospheric chemistry. A key component in this relationship is the volatile organic compound to NO_x_ ratio (VOC:NO_x_). When this ratio increases, it increases the production of secondary organic aerosols, a primary component in PM_2.5_; thus, leading to an increase in PM_2.5_ (Connerton *et al*., 2020; Bekbulat *et al*., 2021). Lastly, PM can also be attributed to residential fuel combustion, waste management, and fugitive dust, which may not have been affected by COVID-19 restrictions (Chen *et al*., 2020).

Increases in O_3_ concentrations have been attributed to the nonlinear photochemical reaction, which involves NO_x_, VOCs, and sunlight (Jin *et al*., 2017). Effects of the precursor concentration production rate can be categorized as either NO_x_ limited, or VOC limited. NO_x_-limited is when the VOC:NO_x_ ratio is large, and the VOC-limited environment happens when the VOC:NO_x_ ratio is smaller. In areas with a VOC-limited regime, a decrease in NO_x_ concentrations will cause an increase in O_3_ production (Chen *et al*., 2020; Fu *et al*., 2020). Most of the studies that observed increases in O_3_ cited this photochemical reaction as an explanation.

### 3.5 Contextual Factors

Twenty articles tried to explain the change of air pollution levels through different contextual factors. Eight studies incorporated mobility data into the analyses. Sources of these mobility data were from Google, Apple, and a dataset developed by Descartes Labs that provides an aggregated mobility measure based on anonymized mobile device locations. Nine articles examined how traffic flow was related to changes in air quality. Traffic flow information was obtained via TRACFLOW (Xiang *et al*., 2020), TomTom traffic congestion data (Liu *et al*., 2020; Winchester *et al*., 2021), Caltrans Performance Measurement System (Liu *et al*., 2021a; Naeger and Murphy, 2020; Parker *et al*., 2020), Transit app (Pan *et al*., 2020), Streetlight VMT (Jia *et al*., 2020), and the Federal Highway Administration (Elshorbany *et al*., 2021). Parameters used in these analyses were total vehicle volume (TOV), road occupancy (%), public transit demand, vehicle miles traveled (VMT), and daily traffic counts. Two studies performed a health assessment of how many prevented premature deaths resulted from reducing air pollution during the COVID-19 restrictions. Finally, two studies examined the disparities of NO_2_ pollution for disproportionally impacted populations across the US (Winchester *et al*., 2021). Their findings revealed the most significant reductions occurred in marginalized areas, but the effect of lockdowns on racial, ethnic, and socioeconomic NO_2_ disparities was mixed and, for many cities, nonsignificant (Kerr *et al*., 2021).

## 4 DISCUSSION

Generally, we saw a decrease in NO_2_ and CO and mixed directions of other air pollutants in response to COVID-19 mitigation strategies, and the effects of pandemic mitigation practices on air pollutants were heterogeneous across the US. This heterogeneity was observed across both large and small spatial scales. Changes in air pollutants were found to differ by and within regions, counties, and cities. This observed heterogeneity highlights the complex interactions between air pollutants, meteorological conditions, pandemic mitigation efforts, geographical location, and other factors that could be driving this array of effects. Studies that examined only metropolitan areas found nonuniform effects of COVID-19 mitigation policies on the same air pollutant. Only a few studies tried to elucidate these nonuniform effects by examining individual mobility and traffic flow patterns.

We identified crucial gaps in the literature through our scoping review, which examined how air quality changed through the span of this scoping review. One such gap that was elucidated related to the dates of publication and the length of study periods. Nearly all studies included in this scoping review encompassed study periods that only covered a portion of 2020. Most of the published articles in 2020 examined changes in air quality only until May of 2020. Furthermore, many studies published in 2021 did not assess the change in air pollution for 2020 in its entirety. These truncations of study periods resulted in missed information about the renewed COVID-19 mitigation strategies implemented because of the significant surge of COVID-19 cases at the end of 2020 in the US, and the emergence and additional practices employed to reduce the spread of the subsequent novel coronavirus variants. Truncated study periods do ignore potential changes to air quality during other times when mitigation strategies were in effect to address the surges of COVID-19 cases, however, the impact of these strategies on air quality changes can be different. For instance, understanding changes in mobility patterns during the initial wave of cases and comparing that to subsequent periods when mitigation strategies were in effect can help quantify impact on relevant air pollution sources.

While mitigation measures have influenced transportation-related emissions, there are other emission sources that could have contributed to an increase in air pollutant concentrations. Most notably, many of these articles only explored air quality changes during the initial stages of the pandemic and did not include time periods when record-breaking wildfires occurred, during the latter part of 2020. We acknowledge that there was an urgency to disseminate findings from these studies, which could have led to the short study periods. However, these short study periods would not allow for ascertainment of contributions from individual sources in areas where air pollution is from multiple sources. Wildfires are a significant source of air pollutant emissions, including PM_2.5_ (Ford *et al*., 2018; Gupta *et al*., 2018; Aguilera *et al*., 2021). These increased emissions of PM_2.5_ could have influenced the findings of studies that examined air pollutant changes throughout 2020, especially in the Western US. The possible effect of excess PM_2.5_ from wildfires was supported by the findings of two articles included in this scoping review that examined air pollution changes until September and December of 2020, which found PM_2.5_ and AOD levels were higher than expected (Bekbulat *et al*., 2021; Elshorbany *et al*., 2021).

The majority of articles included in this scoping review relied upon ground monitors or remote sensing data. We believe the researchers had a good argument to utilize remote sensing data because it gives the opportunity to retrieve near real-time aerosol data (if data from ground monitors are not available), even though calibration and cloud cover can be an issue (Berman and Ebisu, 2020). Although we did not quantitively assess the quality of the articles included in the scoping review, we found that over half the articles utilized basic methodologies that included descriptive studies, correlation studies, and comparison studies calculating percent change. Ideally, a measurement-based study could address some of the limitations identified in these papers. For example, performing a time-series analysis using autoregressive moving averages model adjusted for meteorological and mobility confounders and accounting for seasonality of the pollutants and meteorological variables could be a valid approach. Additionally, articles that examined change in air quality as a function of mobility should have chosen an appropriate data source that addresses the research question at hand, as these data vary by individual and aggregated scale and across various spatial and temporal scales (Hu *et al*., 2021).

A concerning body of evidence suggests that climate hazards such as extreme heat, wildfires, drought, floods, and hurricanes, which are increasing in frequency and intensity in many regions under climate change, intersect with the COVID-19 pandemic and public health response (Phillips *et al*., 2020). Thus, it might be equally important to understand how exposure to these additional climate hazards was affected by pandemic mitigation strategies in the US, as there is growing evidence to suggest that cascading health effects resulting from exposure to compound hazards (e.g., air pollution and extreme heat) is a significant public health concern. Furthermore, evidence from this scoping review, which summarized the relationships between changes in air pollution and mitigation practices, can shed light on how climate adaptation and mitigation efforts aimed at reducing greenhouse gas emissions might influence air quality levels at local and regional levels scales.

There are several limitations to this scoping review. Articles published after the retrieval date were not able to be included in our study. Also, we did not rank the suitability of the methodology or any inherent biases due to the study design implemented for these articles because our main goal was to summarize the change in air quality linked to COVID-19 mitigation strategies. Future research could aim to weigh the methodologies used across these studies differently. Furthermore, due to the numerous studies examining air quality changes within the same geographical area, there were instances where the magnitude of change for air pollutants was reported using different concentration units that were not comparable (e.g., vertical column density [moles m^–2^] versus routinely reported concentration units [ppb]). In such instances, we excluded studies using those non-traditional reporting units from Fig. 3.

## 5 CONCLUSION

Overall, we identified 66 scientific articles that assessed the change in air quality related to COVID-19 mitigation efforts. Evidence from these studies showed nonuniform changes in air pollution at the regional, state, county, and local scales across the US. CO and NO_2_ generally decreased across all geographic locations, O_3_ was found to increase in urban areas and decrease in rural areas, and PM_2.5_ concentrations changes were the most variable across the US. A portion of the studies included in this review attempted to explain the observed heterogeneity in air quality change by examining how various contextual factors, such as mobility, traffic patterns, and socioeconomic status, could be associated and found that traffic-related emissions and mobility decreased the most in early 2020. This scoping review has summarized the available evidence and can be utilized to elucidate further the relationship between pandemic mitigation practices and changes in air quality. Lastly, from a climate change perspective, understanding the relationship between air pollution and contextual factors, such as measures of baseline health equity, behavioral modification due to compounding hazards, changes in mobility patterns as measured before and after a public health intervention, can help inform the effectiveness of policies aimed at reducing greenhouse gas emissions.

## Supplementary Material

Supplementary_AAQR_COVID_AQ

## Figures and Tables

**Table 1.** List of articles included in this scoping review.

Author	Journal	Published date	Study Period	Study Location	Pollutants
Amouei Torkmahalleh *et al*. (2021)	Aerosol and Air Quality Research	1/28/2021	1/1/20204/30/2020	Global	NO_2_, PM_2.5_, O_3_
Naeger and Murphy (2020)	Aerosol and Air Quality Research	8/10/2020	3/9/20204/10/2020	California	NO_2_, PM_2.5_
Shakoor *et al*. (2020)	Air Quality, Atmosphere & Health	8/9/2020	1/18/20203/25/2020	Global	CO, NO_2_, PM_2.5_, PM_10_
Habibi *et al*. (2020)	Atmosphere	11/27/2020	1/1/20204/30/2020	Global	CO, NO_2_, PM_2.5_, O_3_
Fu *et al*. (2020)	Atmosphere	11/3/2020	1/23/20205/10/2020	Global	CO, NO_2_, PM_2.5_, PM_10_, O_3_, SO_2_
Jia *et al*. (2020)	Atmosphere	6/14/2020	3/25/20204/23/2020	Memphis, Tennessee	NO_2_, PM_2.5_, O_3_
Keller *et al*. (2021)	Atmospheric Chemistry and Physics	1/21/2021	1/1/20207/1/2020	Global	NO_2_, O_3_
Jiang *et al*. (2021)	Atmospheric Chemistry and Physics	6/9/2021	2/18/20204/23/2020	Southern California	CO, NH_3_, NO_x_, PM_2.5_, PM_10_, SO_x_
Miech *et al*. (2021)	Atmospheric Environment	4/1/2021	1/6/20204/8/2020	Phoenix, Arizona	CO, NO_2_, PM_10_
Savtchenko and Khayat (2021)	Atmospheric Environment	6/1/2021	10/1/20044/30/2020	Global	NO_2_
Bray *et al*. (2021)	Atmospheric Environment	6/21/2021	1/1/20204/30/2020	Global	CO, NO_2_, PM_2.5_, PM_10_, O_3_, SO_2_
Campbell *et al*. (2021)	Atmospheric Environment	11/1/2021	3/1/20209/30/2020	Continental US	O_3_
Ghosal and Saha (2021)	Atmospheric Environment	6/1/2021	1/1/20206/29/2020	Continental US	PM_2.5_
Archer *et al*. (2020)	Bulletin of Atmospheric Science and Technology	10/26/2020	3/31/20204/30/2020	Continental US	NO_2_, PM_2.5_
Bar *et al*. (2021)	Cities	10/1/2021	3/1/20205/30/2020	Europe and US	NO_2_, PM_2.5_
Balasubramaniam *et al*. (2020)	Energy Sources, Part A: Recovery, Utilization, and Environmental Effects	10/1/2020	3/1/20205/30–2020	US, Italy, and France	NO_2_, PM_2.5_, O_3_
Hoang *et al*. (2021)	Energy Sources, Part A: Recovery, Utilization, and Environmental Effects	3/1/2021	NA	Global	NO_2_
Mousazadeh *et al*. (2021)	Environment, Development, and Sustainability	2/4/2021	NA	Global	CO, NO, NO_2_, PM_2.5_, PM_10_, O_3_, SO_2_
Sokhi *et al*. (2021)	Environment International	12/1/2021	1/1/20209/30–2020	Global	CO, NO, NO_2_, PM_2.5_, PM_10_, O_3_, SO_2_
El-Sayed *et al*. (2021)	Environmental Pollution	9/15/2021	3/15/20204/15/2020	Florida	CO, NO_2_, PM_2.5_, O_3_, SO_2_
Acharya *et al*. (2021)	Environmental Research	2/1/2021	3/22/20204/30/2020	Southeast Asia, Europe, and the US	AOD, NO_2_, SO_2_
Sannigrahi *et al*. (2021)	Environmental Research	3/4/2021	2/1/20205/11/2020	Global	CO, NO_2_, PM_2.5_, PM_10_
Venter *et al*. (2021)	Environmental Research	1/1/2021	1/1/2020–5/15/2020	Global	NO_2_, PM_2.5_, O_3_
Mendoza *et al*. (2021)	Environmental Research	10/1/2021	2/3/2020–7/23/2020	Park City, Utah	PM_2.5_
Chauhan and Singh (2020)	Environmental Research	8/1/2020	12/1/2019–3/31/2020	Global	PM_2.5_
Wong *et al*. (2021)	Environmental Research Letters	5/11/2021	2/15/2020–5/6/2020	Continental US	NO_2_
Shehzad *et al*. (2021)	Environmental Science and Pollution Research	3/29/2021	1/1/2020–5/3/2020	New York City, New York	CO, NO_2_, PM_2.5_, O_3_, SO_2_
Liu *et al*. (2021a)	Environmental Science and Technology Letters	12/4/2020	1/1/2020–4/30/2020	California	NO, NO_2_
Tanzer-Gruener *et al*. (2020)	Environmental Science and Technology Letters	6/1/2020	3/1/2020–3/31/2020	Pittsburg, PA	CO, NO_2_, PM_2.5_
Bauwens *et al*. (2020)	Geophysical Research Letters	6/16/2020	1/1/2020–4/30/2020	Global	NO_2_
Turner *et al*. (2020)	Geophysical Research Letters	10/30/2020	2/2/2020–5/2/2020	San Francisco, CA	CO_2_
Parker *et al*. (2020)	Geophysical Research Letters	11/1/2020	3/19/2020–6/30/2020	South Coast Air Basin (SoCAB), California	NO_2_, NO_x_, PM_2.5_, O_3_
Goldberg *et al*. (2020)	Geophysical Research Letters	8/17/2020	1/1/2020–4/30/2020	Continental US	NO_2_
Irfan *et al*. (2021)	International Journal of Environmental Research	1/1/2021	NA	China, USA, Italy, and Spain	NO_2_
Connerton *et al*. (2020)	International Journal of Environmental Research and Public Health	7/14/2020	3/1/2020–3/31/2020	São Paulo, Brazil; Paris, France; Los Angeles, California; and New York	CO, NO_2_, PM_2.5_, O_3_
Chadwick *et al*. (2021)	Journal of Aerosol Science	6/1/2021	2/11/2020–5/11/2020	Salt Lake City, Utah	PM_2.5_
Sahraei *et al*. (2021)	Journal of Environmental Management	5/1/2021	1/15/2020–5/31/2020	Global	CO, NO_2_, PM_2.5_, PM_10_, O_3_, SO_2_
Dang *et al*. (2021)	Journal of Environmental Economics and Management	1/1/2021	1/1/2020–3/31/2020	Global	NO_2_, PM_2.5_
Brodeur *et al*. (2021)	Journal of Environmental Economics and Management	3/1/2020	3/19/2020–5/31/2020	Continental US	PM_2.5_
Kondragunta *et al*. (2021)	Journal of Geophysical Research: Atmosphere	8/17/2021	2/11/2020–5/11/2020	Continental US	NO_x_
Liu *et al*. (2020)	Nature Communications	10/14/2020	1/1/2020–6/30/2020	Global	CO_2_
Kerr *et al*. (2021)	Proceedings of the National Academy of Sciences	8/19/2021	3/13/2020–6/13/2020	Continental US	NO_2_
Venter *et al*. (2020)	Proceedings of the National Academy of Sciences	7/28/2020	1/1/2020–5/31/2020	Global	AOD, NO_2_, O_3_
Straka *et al*. (2021)	Remote Sensing	12/22/2020	2/1/2020–4/30/2020	Los Angeles, California; Chicago, Illinois; Washington DC	NO_2_
Zhang *et al*. (2020)	Remote Sensing	7/1/2020	1/1/2020–5/15/2020	Global	AOD, CO, NO_2_, PM_2.5_, O_3_, SO_2_
Elshorbany *et al*. (2021)	Remote Sensing	1/1/2021	1/1/2020–12/31/2020	New York, Illinois, Florida, Texas, and California	AOD, CO, NO_2_, O_3_
Miyazaki *et al*. (2021)	Science Advances	6/9/2021	2/1/2020–7/31/2020	Global	NO_x_, O_3_
Hammer *et al*. (2021)	Science Advances	6/23/2021	1/1/2020–4/30/2020	Global	PM_2.5_
Chossiere *et al*. (2021)	Science Advances	5/21/2021	1/1/2020–7/6/2020	Global	NO_2_, PM_2.5_, O_3_
Liu *et al*. (2021c)	Science of the Total Environment	7/1/2021	1/1/2020–6/30/2020	China, USA, UK, Brazil, South Africa, and India	NO_2_
Zhang *et al*. (2021)	Science of the Total Environment	3/20/2021	1/1/2020–4/30/2020	Australia, Canada, China, and United States	NO_2_
Liu *et al*. (2021b)	Science of the Total Environment	1/1/2021	1/26/2020–6/14/2020	California	CO, NO_2_, PM_2.5_, PM_10_, O_3_, SO_2_
Muhammad *et al*. (2020)	Science of the Total Environment	8/1/2020	NA	Global	NO_2_
Mishra *et al*. (2021)	Science of the Total Environment	8/15/2021	3/24/2020–6/15/2020	India, China, France, Brazil and United States	PM_2.5_
Zangari *et al*. (2020)	Science of the Total Environment	11/1/2020	1/1/2020–5/30/2020	New York	NO_2_, PM_2.5_
Xiang *et al*. (2020)	Science of the Total Environment	12/1/2020	2/17/2020–3/31/2020	Seattle, Washington	BC, CO, NO, NO_2_, NO_x_, PM_2.5_
Chen *et al*. (2020)	Science of the Total Environment	7/21/2020	1/1/2020–4/30/2020	Continental US	CO, NO_2_, PM_2.5_, PM_10_, O_3_
Zambrano-Monserrate *et al*. (2020)	Science of the Total Environment	8/1/2020	NA	China, USA, Italy, and Spain	NO_2_
Berman and Ebisu (2020)	Science of the Total Environment	10/1/2020	1/8/2020–4/21/2020	Continental US	NO_2_, PM_2.5_
Hudda *et al*. (2020)	Science of the Total Environment	11/10/2020	3/27/2020–5/14/2020	Somerville, Massachusetts	BC, PNC
Bekbulat *et al*. (2021)	Science of the Total Environment	5/15/2020	1/1/2020–10/1/2020	Continental US	CO, NO_2_, PM_2.5_, PM_10_, O_3_
Heintzelman *et al*. (2021)	Sustainability	8/12/2021	3/1/2020–4/30/2020	Continental US	NO_2_
Winchester *et al*. (2021)	Sustainability	6/29/2021	1/1/2020–8/31/2020	Continental US	NO_2_
Pan *et al*. (2020)	Sustainability	8/1/2020	3/20/2020–5/30/2020	California	PM_2.5_, O_3_
Yusup *et al*. (2020)	Sustainability	11/1/2020	3/1/2020–5/31/2020	China, the United States, Europe, and India	CO_2_
Wang and Li (2021)	Sustainable Cities and Society	2/1/2021	1/23/2020–10/30/2020	Wuhan, New York, Milan, Madrid, Bandra, London, Tokyo and Mexico City	NO_2_, PM_2.5_, O_3_, SO_2_
